# Two-stage association study of mitochondrial DNA variants in allergic rhinitis

**DOI:** 10.1186/s13223-024-00881-z

**Published:** 2024-02-23

**Authors:** Huajie Yuan, Lingling Wang, Song Wang, Linge Li, Qingping Liu, Yan Wang, Yuping Yang, Hua Zhang

**Affiliations:** Department of Otolaryngology, Xinjiang Medical University Affiliated First Hospital, 137 Liyushan Avenue, Xinshi District, Urumqi, 830054 Xinjiang China

**Keywords:** Allergic rhinitis, Mitochondrial, Gene, Variants

## Abstract

**Background:**

Correlations between mitochondrial DNA (mtDNA) and allergic rhinitis (AR) have not been reported before. This study aimed to better understand the mitochondrial genome profile with AR and to investigate the associations between AR in China and the mitochondrial genome at a single variant and gene level.

**Methods:**

Mitochondrial sequencing was conducted on a total of 134 unrelated individual subjects (68 patients with AR, 66 healthy controls) at discovery stage. Heteroplasmy was analyzed using the Mann-Whitney U test. Sequence kernel association tests (SKAT) were conducted to study the association between mitochondrial genes and AR. Single-variant analysis was performed using logistic regression analysis and further validated in 120 subjects (69 patients with AR, 51 healthy controls). Candidate genes were further explored based on differences in mRNA and protein abundance in nasal mucosal tissue.

**Results:**

In the discovery stage, 886 variants, including 836 SNV and 50 indels, were identified with mitochondrial sequencing. No statistically significant differences were identified for the mitochondrial heteroplasmy or SKAT analysis between these two groups after applying a Boferroni correction. One nonsynonymous variants, rs3135028 (MT8584.G/A) in ATP6, was related to a reduced risk of AR in both the discovery and validation cohorts. Furthermore, mRNA levels of MT-ATP6 in nasal mucosal tissue were significantly lower in AR individuals than in controls (P < 0.05).

**Conclusions:**

In a two-stage analysis of associations between AR and mtDNA variations, mitochondrial gene ma*p*s of Chinese patients with AR indicated that the ATP6 gene was probably associated with AR at the single-variant level.

**Supplementary Information:**

The online version contains supplementary material available at 10.1186/s13223-024-00881-z.

## Background

Allergic rhinitis (AR) is the most prevalent atopic disease in the world, affecting up to 40% of adults and 25% of children [1]. Although AR is typically a mild disease, it dramatically impairs patients' social lives and performance at school and work. Furthermore, the high incidence of rhinitis comes at a substantial financial expense. Additionally, one of the common reasons for going to primary care clinics is AR, which has often been linked to asthma, sinusitis, nasal polyposis, otitis media, and conjunctivitis [2].

Although the exact cause of AR is unknown, genetic-environmental interactions play a role in its occurrence [3, 4]. According to twin studies in Europe, the estimated heritability of AR ranges between 33 and 91% [5], implying that genetic risk factors likely significantly influence the disease. Candidate gene studies, genome-wide analyses, and more recent studies integrating gene expression with epigenetic alterations have all been used to discover genes associated with AR risk. These studies add functional information to DNA sequence variants. The understanding of AR pathophysiology has improved thanks to the development of such potent new genetic research techniques [6].

When cells acquire internal and external stimuli, mitochondria play a critical role in the decision-making process, leading to various biological consequences, such as metabolic adaptation, proliferation, differentiation, and cell death [7]. Meanwhile, variants in mitochondrial DNA (mtDNA) are believed to be associated with allergic illness [8]. The results showed that mitochondrial dysfunction led to antigen-driven allergic airway inflammation, and it could be a risk factor for the increase of allergic airway inflammation [9]. As a complex human disease, AR is an example of an allergic illness that has been linked to mitochondrial function (MT-nDNA) resulting from mtDNA and nuclear DNA variations [6, 10]. It is known that altered mitochondrial function is caused by mitochondrial mutation on the one hand, and related to the copy number of mitochondrial DNA on the other. Previous studies by our research group have found that the copy number of mitochondrial DNA in peripheral blood of AR is significantly higher than that of patients without AR [11]. In terms of mtDNA, mitochondrial dysfunction might be the second hit to AR inflammatory reaction caused by mitochondrial genes and metabolic pathways. Li et al. highlighted several susceptibility loci for AR in a review of genome-wide association studies (GWAS) performed in patients with this condition [12]. The mitochondrial 39S ribosomal protein L4, encoded by the MRPL4 gene, is hypothesized to contribute to ribosomal integrity and mitochondrial protein translation [13]. Considering common variants from GWAS explain only a fraction of the genetic heritability of AR, Bunyavanich et al. used CD4 + lymphocytes (CD4 cells) isolated from the peripheral blood of 5,633 ethnically diverse North American individuals to perform integrative investigations that linked genetic variations with gene expression. Using this approach, the authors demonstrated the significance of mitochondrial pathways in the pathophysiology of AR [14]. There is also accumulating evidence that mitochondrial signaling and metabolism are necessary to establish and regulate various immunophenotypes, including CD4 T-cell differentiation [15]. Interestingly, these two studies have linked mitochondria to the pathophysiology of AR.

Pathogenic point mutations and large deletions with heterogeneity in mtDNA are found using mitochondrial DNA whole genome sequencing (m-WGS) in a non-biased approach. Mitochondrial DNA variants resulting in mitochondrial disruption or metabolic imbalance, which induces allergic diseases, such as asthma [16]. However, the contribution of mitochondrial whole genome sequencing technology to the genetics of AR has yet to be explored.

In this study, variants in mtDNA associated with mitochondrial function were examined using m-WGS to determine variant-based and gene-based associations with AR. In addition, the heterogeneity of mtDNA was studied in patients with AR.

## Methods

### Study subjects

For this case–control study, 134 unrelated individual subjects (68 patients with AR, 66 healthy controls) at the discovery stage were enrolled from the outpatient clinic of the First Affiliated Hospital of Xinjiang Medical University from December 2017 to March 2018. In the second cohort, 120 subjects (69 patients with AR, 51 without AR) were recruited from the otolaryngology ward between May 2019 and December 2019. Patients with AR combined with a deviated nasal septum were considered case patients, and those with a deviated nasal septum without AR were selected as the control group. In addition, nasal mucosa tissue samples from 24 patients from the validation cohort (18 patients for mRNA level analysis and 6 patients for protein level analysis) were collected again to study the expression of mitochondrial mutant genes.

There were similar living environments and lifestyle habits of all Han Chinese subjects in the discovery and validation cohorts over three or more years before enrollment within the Xinjiang Uygur Autonomous Region of China.

### Clinical diagnoses

Patients in the case group had a history of AR symptoms for more than two years based on the 2015 Chinese Guidelines for the Diagnosis and Treatment of Allergic Rhinitis [17]. Other inclusion criteria included: (1) suffering from two or more AR symptoms, including sneezing, rhinorrhea, nasal itching, and nasal blockage, that continued for at least an hour each day, which could be accompanied by other symptoms, such as itchy eyes, tears, red eyes and other eye symptoms; (2) signs including watery nasal discharge, edema and pale nasal mucosa, conjunctival edema, and congestion; and (3) a positive allergen test comprised of a minimum of one skin prick test (SPT) or a positive serum-specific IgE test result. The inclusion criteria of the subjects in the control group are mentioned below: a clinical history of AR, anterior rhinoscopy and/or nasal endoscopy outcome(s), allergen detection and serum-specific IgE test should all be negative. AR diagnoses were made by specially-trained practitioners. None of the subjects experienced malignancy or a serious allergic reaction, underwent an organ transplant, or had liver or kidney failure. Pregnant women and people with immune deficiencies were not included in this study.

### Data collection

Age, sex, family history of AR, history of drug allergies, and other clinical data were extracted from medical records. SPT, serum-specific IgE, and anterior rhinoscopy or nasal endoscopy outcome(s) were recorded and examined. Allergen SPT positivity was defined as 3 mm of growth in a weal pattern or a minor swelling of the skin within 15–20 min of being pricked with histamine versus a negative control using a 0.9% saline solution. Serum-specific IgE positivity was defined as IgE levels ≥ 0.35 kU*/*L [18]. Venous blood (3 mL) from each individual was collected into ethylenediaminetetraacetic acid (EDTA) test tubes, then temporarily stored at − 80 °C until the next steps. In consideration of invasiveness and the impact of the COVID*-*19 pandemic, 24 patients (18 patients for mRNA level analysis and 6 patients for protein level analysis) in the validation cohort with deviated nasal septums who were awaiting surgery were selected for nasal mucosal tissue collection to minimize trauma to the patients. At the same time, fresh tissues that the electric knife had not cauterized were collected as often as possible to ensure the availability of tissue specimens. The nasal mucosa was clamped and cleaned during the operation and quickly dispensed into 5 mL lyophilization tubes to avoid exposing the tissues to air for too long, then put into liquid nitrogen tanks.

### m-WGS and analysis

Genomic DNA was isolated from EDTA anti-coagulated venous blood samples using the Wizard Genomic DNA Purification Kit (Promega, Beijing, China), following the manufacturer’s instructions. The DNA samples were determined by electrophoresis and quantified using NanoDrop 2000. Shanghai Genesky Biotechnologies captured the mitochondrial genome. Long-range PCR was designed to amplify the mtDNA using specific primers for the human mitochondrial genome. Agencourt AMPure XP-PCR Purification kits (Beckman, Germany) were used to pool and purify the six partially overlapping PCR products, each measuring roughly 5 kb. After that, ultrasonography was used to fragment the PCR results into 100–500 bp fragments (ME220, Woburn, MA). The NEBNext DNA Library Prep Reagent Set from Illumina (New England Biolabs, Ipswich, MA) was used to perform end-repairing, A-tailing, and adaptor ligation after DNA fragmentation. Following PCR amplification, the amplicons were again analyzed and quantified using the BioAnalyzer 2100 before being submitted to 2 × 150 bp paired-end massively parallel sequencing on an Illumina NovaSeq System (Illumina).

The sequencing of human mitochondrial genome is to observe the mutation in individual mitochondrial genomes by double-ended sequencing of the whole mitochondrial genome sequence of each sample. FastQC raw files(version0.11.5) were collected for the sequencing quality evaluation, including the base mass distribution and base composition distribution of each sample. The sequences were aligned against the mitochondrial reference data (revised Cambridge Reference Sequence, rCRS), and sequence alignment files were generated by the Burroughs-Wheeler Aligner (BWA). Variant calling was performed utilizing Mutect2 and Haplotype Caller from the Genome Analysis Toolkit (GATK). Further, variations were sorted based on the threshold (mapping quality 20 and base quality 20). Then, a mitochondrial DNA library was constructed and the length distribution of inserted fragments was counted. The average coverage depth of the genome sequence of samples in this study exceeded 100X, so it is considered that the SNV detected at this site is relatively reliable. Otherwise, variants with less 100X coverage were deleted or re-tested. Variants with frequency levels between 0.1 and 0.9 were regarded as heteroplasmic. The average number of distinct heteroplasmic variations found in the designated site across all samples within the group was used to calculate the number of mtDNA variants.

### SNaPshot analysis

The candidate single nucleotide polymorphisms (SNP) loci were subjected to the SNaPshot SNP test for genotyping. The sequences of the PCR primer pairs utilized to amplify sequences of genomic DNA are described in Additional file 1: Table S1. An ABI3130XL sequencer and GeneMapper 4.0 software (Applied Biosystems, Co. Ltd., USA) were used to analyze the collected data. To confirm the accuracy of genotyping quality, random samples of 5% of cases and controls were genotyped twice by blinded laboratory personnel. The results of the repeated samples were more than 99% similar.

### qRT-PCR validation

Total RNA was extracted from frozen nasal mucosal tissue samples stored at − 80 ℃ using Trizol reagent (Invitrogen). One ug of RNA was reverse-transcribed using random primers, and one ul of the resultant single-stranded cDNA was used as the template. The qRT-PCR assay was designed using a SYBR-Green PCR kit and analyzed in triplicate using the Applied Biosystems 7900HT Fast Real-Time PCR System (Applied Biosystems, Foster City, CA, USA). The primers used for the qRT-PCR assay are detailed in Additional file 2: Table S2. The 2ΔCt formula was used to calculate the outcome data.

### Western blotting

The Total Protein Extraction (TPE) kit (Sangon Biotech) was used in accordance with the manufacturer’s instructions to extract total protein from nasal mucosal frozen tissue samples stored at − 80 °C. As previously described [19], proteins were separated using sodium dodecyl sulfate–polyacrylamide gel electrophoresis and transferred onto polyvinylidene difluoride (PVDF) membranes (EMD Millipore, Billerica, Massachusetts, USA). PVDF membrane was exposed using an enhanced chemiluminescence detection method after being blocked with non-fat milk and incubated with primary and secondary antibodies. The bands' intermixed density was measured using Image Lab software (Bio-Rad, Hercules, CA, USA). Anti-MT- ATP6 (CPA1767) was purchased from Cohesionbio, and anti-GAPDH antibody (8245) from Abcam.

### Ethics statement

The study was approved by the medical ethics committee of the First Affiliated Hospital of Xinjiang Medical University (20,170,316–07), and all participants signed informed consent.

### Statistical analysis

Normally distributed data for continuous variables are described as means with standard deviations, and the two groups were compared by t-test. Non-normal distribution data are presented as the median (interquartile range, IQR), and groups were analyzed using the Mann*–*Whitney U test. The heteroplasmic level between AR patients and controls was determined using the Mann–Whitney U test if at least three samples from each group were carriers. Count data were expressed as percentages, and the two groups were compared using the Chi-squared test. R statistical software (version 3.6.2) was used to evaluate all statistics. MtSNPs with minor allele frequency above 0.05 were used for single variant association analysis. Using the sequence kernel association test provided by the R package SKAT, mitochondrial gene set-based analyses were performed using mtSNV with a frequency range of 0.1–0.9 and call rate above 0.9. All bilateral tests were based on a P < 0.05, which suggested that the difference between groups was statistically significant.

## Results

### Spectrum of mitochondrial genome variants

Whole mtDNA sequencing was carried out at the discovery stage in 68 patients with AR (34 men and 34 women, mean age of 35.87 ± 13.92 years) and 66 controls (33 men and 33 women, mean age of 37.59 ± 17.64 years) to explore the mitochondrial genomic profile and the contribution of mtDNA variations to AR predisposition. The age and gender distribution showed no statistically significant differences between the groups (P > 0.05). The basic characteristics of all enrolled subjects are listed in Table 1.

**Table 1:** Characteristics of study participants

Variables	Total (n = 134)	Case (n = 68)	Control (n = 66)	*p*-value
Age, Mean (SD)	36.72 (15.82)	35.87 (13.92)	37.59 (17.64)	0.531
Sex, n (%)				0.999
Male	67 (50)	34 (50)	33 (50)	
Female	67 (50)	34 (50)	33 (50)	
IgE, median (Q1,Q3)	42.15 (16.75, 134.75)	115 (41.15, 343)	19.1 (8.17, 45.07)	< 0.001
Family history, n (%)				< 0.001
No	113 (84.33)	47 (69.12)	66 (100)	
Yes	21 (15.67)	21 (30.88)	0 (0)	
Drug allergy, n (%)				< 0.001
No	121 (90.3)	55 (80.88)	66 (100)	
Yes	13 (9.7)	13 (19.12)	0 (0)	

**Fig. 1:**
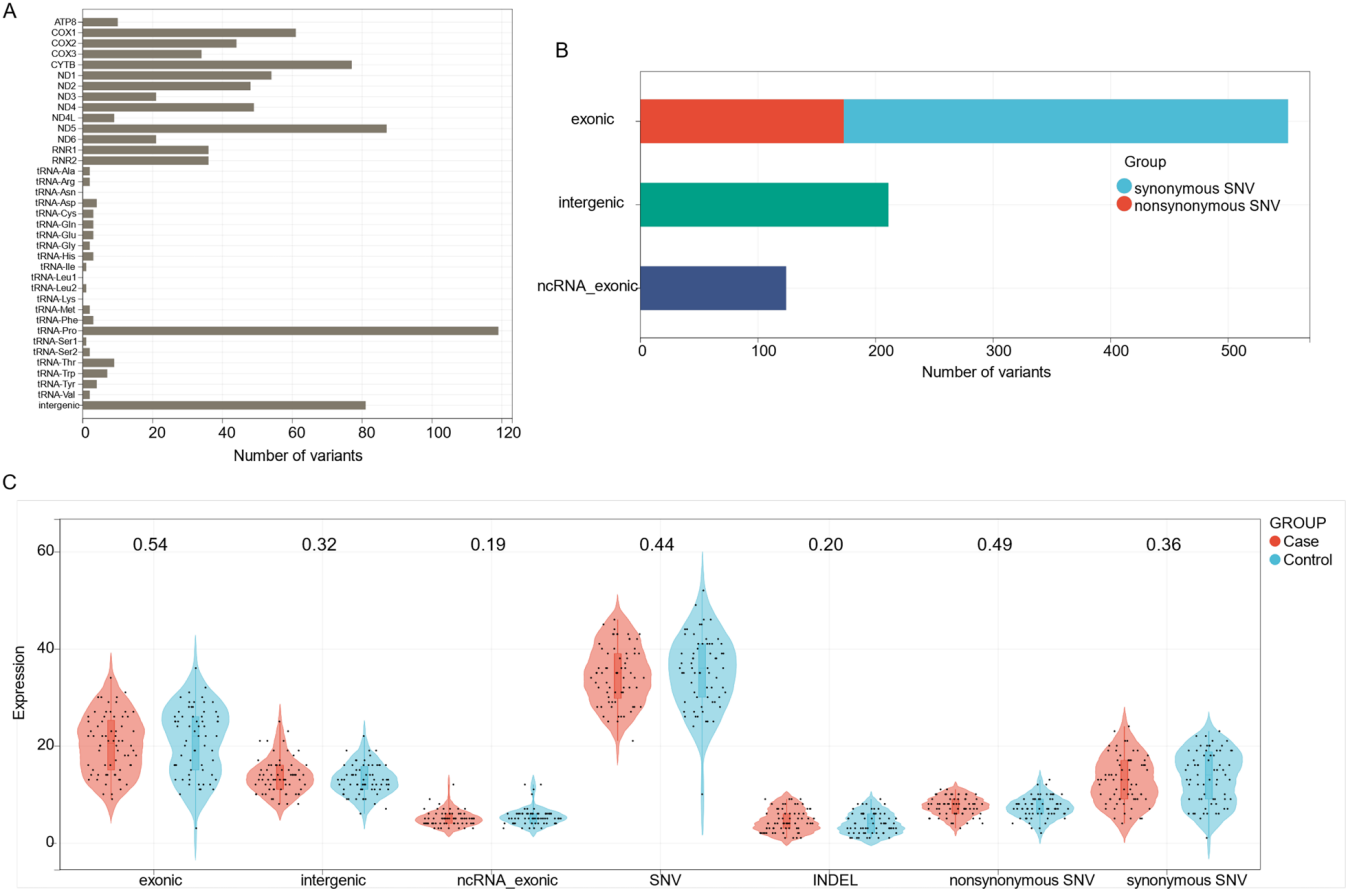
Spectrum of mitochondrial genome variants. **A** The distribution of the variants identified on 37 genes of the mitochondria genome in the discovery cohort. **B** The distribution of the variants identified according to their functional effects. **C** No statistically significant differences were found by comparing the number of variants categorized according to their location, type and effects between the AR and control groups

**Fig. 2:**
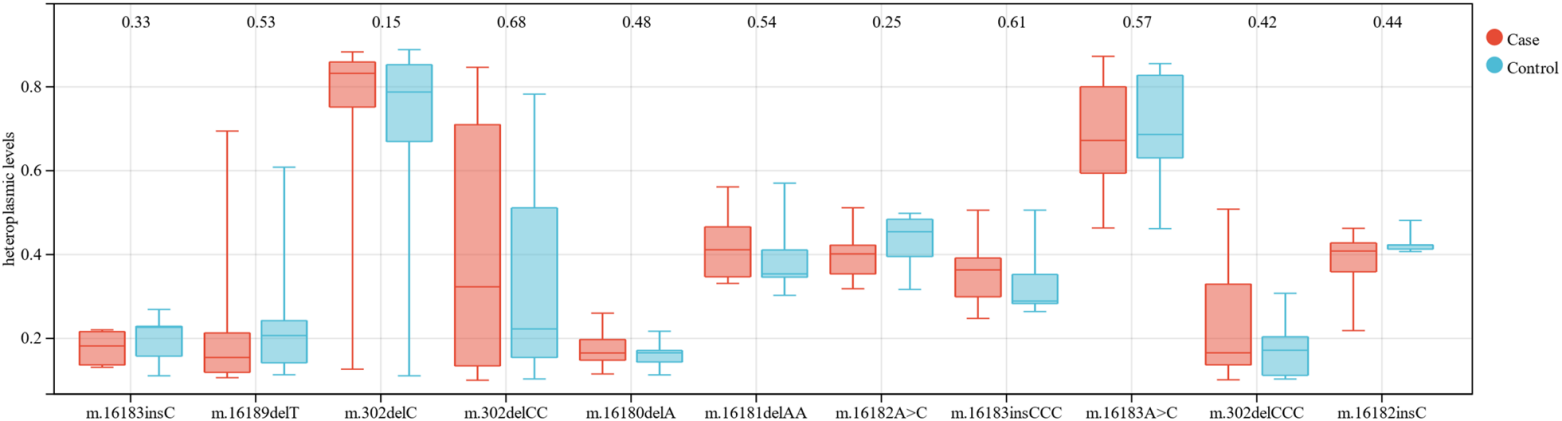
Comparing heteroplasmic variants between groups

Overall, 886 variants were identified in the study population, including 836 SNVs and 50 Indels. The distribution of the variants identified on 37 mitochondrial genes is presented in Fig. 1A. The mitochondrial tRNA (Pro) gene (tRNA-Pro) had the highest number of variations (119), followed by the protein-coding gene ND5 (87 variants, OMIM:516,005; Gene ID: 4540), intergenic region (81 variants), CYTB (77 variants, OMIM: 516,020; Gene ID: 4519), and COX1 (61 variants, OMIM: 516,030; Gene ID:4512). A total of 551 variants were found in the protein-coding region when all the variants were categorized based on their functions, including 378 synonymous and 173 nonsynonymous variants (Fig. 1B). No statistically significant differences in the number of variants were noted when categorized according to their location, type, and effects between the AR and control groups (Fig. 1C). Eleven heteroplasmic variants were compared between the groups, and no statistically significant differences were identified (Fig. 2).

### Association of mitochondrial variants with AR

**Fig. 3:**
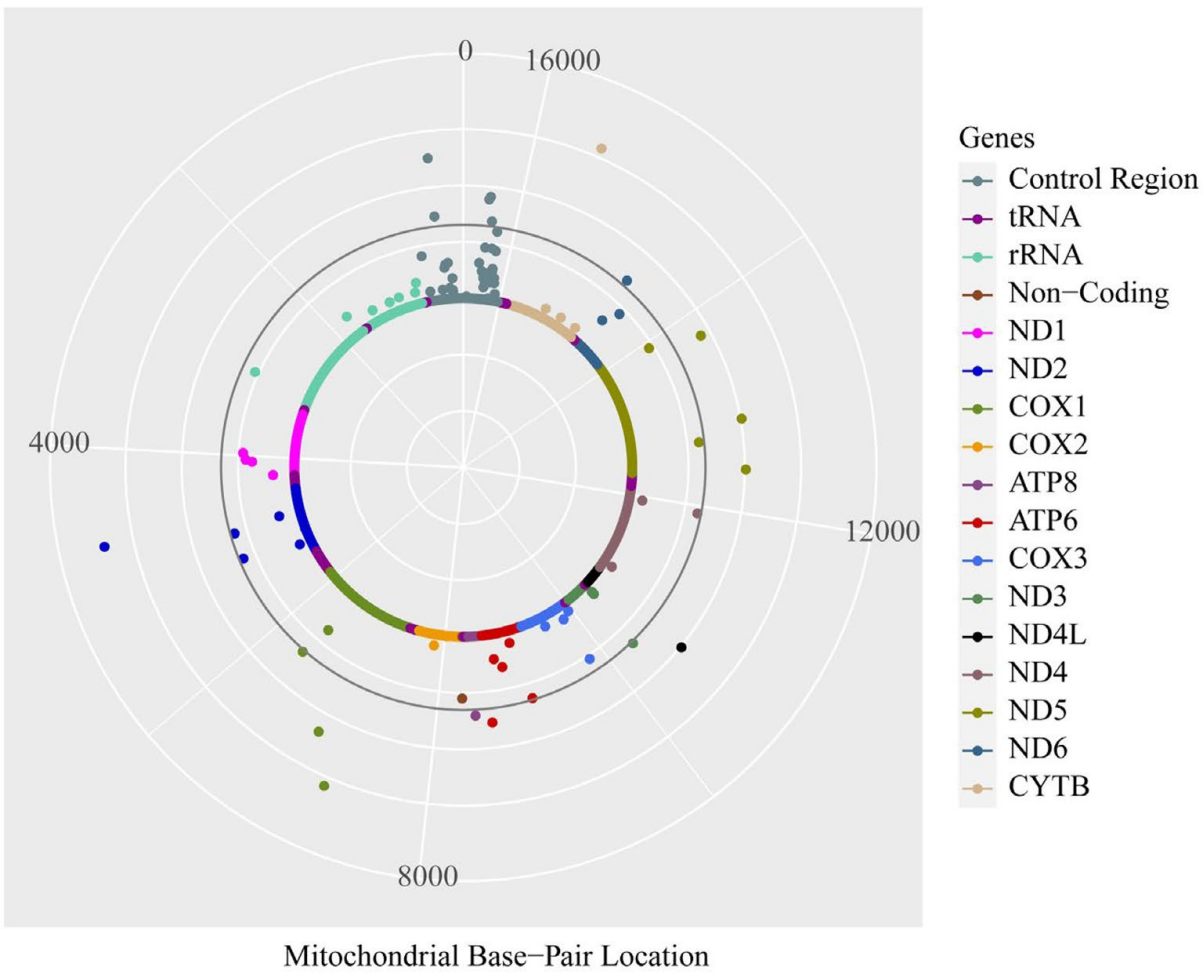
Mitochondrial Manhattan Plot for single variant association analysis of AR. From outside to inside, the three light grey circles correspond to nominal *p* value of 0.001, 0.01 and 0.1. The black circle represents a nominal *p*-value of 0.05. Each dot represents the mtSNP association *p*-value with AR, color-coded by mitochondrial gene

Logistic regression of single variant analysis with the allelic model frequency above 0.05 revealed that 17 of the 92 mtSNPs were associated with AR susceptibility (Fig. 3, Table 2).

**Table 2:** Association analysis of mtSNP in AR group and control group

SNP NO	Gene	Region	Rare Allele	FA	FC	OR (95%CI)	*p*-value	*p*_BH_	*p*_B_
MT4715.A/G	ND2	Synonymous	G	4/132	24/108	0.1332(0.04467–0.3973)	0.0003	**0.0276**	**0.0276**
MT15487.A/T	CYTB	Synonymous	T	4/132	22/110	0.1494(0.04983–0.4477)	0.000687	**0.0316**	0.0632
MT7196.C/A	COX1	Synonymous	A	4/132	22/110	0.1494(0.04983–0.4477)	0.000687	**0.0211**	0.0632
MT302.-/CCC	NONE	Intergenic	CCC	34/102	14/118	2.795(1.417–5.516)	0.003037	0.0699	0.2794
MT6962.G/A	COX1	Synonymous	A	18/118	4/128	5.071(1.652–15.56)	0.004547	0.0837	0.4183
MT10609.T/C	ND4L	Nonsynonymous	C	16/120	4/128	4.5(1.442–14.04)	0.009597	0.1472	0.8829
MT12406.G/A	ND5	Nonsynonymous	A	16/120	4/128	4.5(1.442–14.04)	0.009597	0.1261	0.8829
MT12882.C/T	ND5	Synonymous	T	16/120	4/128	4.5(1.442–14.04)	0.009597	0.1104	0.8829
MT16298.T/C	tRNA-Pro	Intergenic	C	8/128	20/112	0.3442(0.1457–0.8133)	0.01506	0.1111	1.0000
MT13759.G/A	ND5	Nonsynonymous	A	12/124	2/130	6.684(1.445–30.92)	0.01507	0.1000	1.0000
MT16311.T/C	tRNA-Pro	Intergenic	C	26/110	12/120	2.478(1.178–5.213)	0.01682	0.0909	1.0000
MT8584.G/A	ATP6	Nonsynonymous	A	14/122	26/106	0.4546(0.225–0.9186)	0.02805	0.0833	1.0000
MT14668.C/T	ND6	Synonymous	T	34/102	20/112	1.925(1.033–3.587)	0.03914	0.0769	1.0000
MT8414.C/T	ATP8	Nonsynonymous	T	34/102	20/112	1.925(1.033–3.587)	0.03914	0.0714	1.0000
MT16260.C/T	tRNA-Pro	Intergenic	T	4/132	12/120	0.2953(0.09186–0.949)	0.04058	0.0667	1.0000
MT10310.G/A	ND3	Synonymous	A	18/118	8/124	2.461(1.015–5.964)	0.04618	0.0625	1.0000
MT6392.T/C	COX1	Synonymous	C	18/118	8/124	2.461(1.015–5.964)	0.04618	0.0588	1.0000

SNP, single nucleotide polymorphism; MT, mitochondrial; FA, frequency of affected; FC, frequency of control; OR, odd ratio. CI, confidence interval;

*p*_BH,_ *p*_Bonjamini-Hochberg;_ *p*_B,_ *p*_Bofferoni_

Nonsynonymous variants of protein*-*encoding genes and variants in RNA genes were further probed for gene-based associations. The results revealed that the RNR2 and ND6 genes were significantly correlated with AR (P = 0.013 and 0.023, respectively; Additional file 4: Fig. S1). Thirty-sixth mtSNVs in the RNR2 gene and seven variants in the ND6 gene were used for the SKAT analysis (Additional file 4: Fig. S1). However, neither of them survived multiple correction analyses.

### Independent cohort validation for mtSNPs

A total of 120 surgical patients were included in the validation cohort, including 69 patients with AR and 51 non-AR patients. The basic characteristics of all enrolled subjects are listed in Table 3.

**Table 3:** Basic characteristics of all subjects in validation cohort

Variables	Total (n = 120)	Case (n = 69)	Control (n = 51)	*p*-value
Age, median (Q1,Q3)	33 (24, 46)	35 (24, 46)	31 (22, 46)	0.294
Sex, n (%)				0.860
Male	67 (56%)	39 (57%)	28 (55%)	
Female	53 (44%)	30 (43%)	23 (45%)	
IgE, median (Q1,Q3)	53 (16, 190)	148 (45, 299)	16 (8, 29)	< 0.001
Family history, n (%)				< 0.001
No	81 (68%)	30 (43%)	51 (100%)	
Yes	39 (32%)	39 (57%)	0 (0%)	
Drug allergy, n (%)				0.003
No	98 (82%)	51 (74%)	47 (92.16%)	
Yes	21 (18%)	18 (26%)	4 (7.84%)	

Sixteen mtSNPs associated with AR risk with nominal P values below 0.05 were further validated in an independent Chinese Han cohort using the SNaPshot technique. MT302.-/CC was excluded due to test failure. The basic characteristics of all subjects in the validation cohort are described in Table 4. Among these variants, rs3135028 (MT8584.G/A) was associated with AR (P = 0.027). The G allele of rs3135028 in the exonic region of ATP6 decreased the risk of AR, with OR of 0.347(0.133–0.905) compared with the C allele. For the other SNPs, no significant differences in distribution frequencies were identified between the groups (Table 4).

**Table 4:** Allele distribution of mitochondrial SNP in validation cohort

SNP NO	Gene	Genotype	Case	Control	χ^2^	OR (95%CI)	*p*-value
rs148377232 (MT16298.T/C)	tRNA-Pro	C	8	10	1.722	0.512(0.186–1.408)	0.190
		T	61	39			
rs1970771 (MT6962.G/A)	COX1	A	12	9	0.001	0.983(0.379–2.545)	0.971
		G	57	42			
rs200487531 (MT10609.T/C)	ND4L	C	12	9	0.007	0.959(0.370–2.487)	0.932
		T	57	41			
rs28357370 (MT15487.A/T)	CYTB	T	7	7	0.365	0.710(0.232–2.168)	0.546
		A	62	44			
rs28357678 (MT14668.C/T)	ND6	T	12	5	1.464	1.971(0.647–6.005)	0.226
		C	56	46			
rs28357976 (MT4715.A/G)	ND2	G	7	7	0.415	0.694(0.227–2.120)	0.519
		A	62	43			
rs28358875 (MT7196.C/A)	COX1	A	7	7	0.365	0.710(0.232–2.168)	0.546
		C	62	44			
rs28358884 (MT8414.C/T)	ATP8	T	12	5	1.388	1.937(0.636–5.896)	0.239
		C	57	46			
		G	51	39			
rs28617389 (MT12406.G/A)	ND5	A	14	9	0.132	1.188(0.469–3.007)	0.716
		G	55	42			
		T	48	32			
rs3135028 (MT8584.G/A)	ATP6	A	8	14	4.925	0.347(0.133–0.905)	**0.027**
		G	61	37			
rs34799580 (MT16311.T/C)	tRNA-Pro	C	10	7	0.030	1.084(0.382–3.073)	0.880
		T	58	44			
		G	69	48			
rs373855397 (MT16260.C/T)	tRNA-Pro	T	3	2	0.013	1.114(0.179–6.921)	0.908
		C	66	49			
rs376513041 (MT6392.T/C)	COX1	G	15	12	0.036	0.920(0.388–2.183)	0.850
		T	53	39			
		C	69	48			
		A	65	47			
rs386420001 (MT12882.C/T)	ND5	T	12	9	0.001	0.983(0.379–2.545)	0.980
		C	57	42			
		G	63	46			
rs386420024 (MT13759.G/A)	ND5	A	11	7	0.136	1.213(0.435–3.384)	0.712
		G	57	44			
		A	68	48			
rs41467651 (MT10310.G/A)	ND3	T	15	12	0.084	0.880(0.370–2.089)	0.771
		G	54	38			

SNP, single nucleotide polymorphism; MT, mitochondrial; OR, odd ratio; CI, confidence interval

### Candidate analysis based on differences in mRNA and protein abundance

Since rs3135028 (MT8584.G/A) in ATP6 has been successfully verified in the validation stage, ATP6 genes were selected for further candidates analysis based on differences in mRNA and protein abundance between AR patients and healthy control individuals. Nine patients with AR combined with deviated nasal septum were screened as the case group, and nine patients with deviated nasal septum without AR were selected as the control group. Each group contained five males and four females. The mean age of the case group was 30.89 years, while the mean age of the control group was 38.67 years.

**Fig. 4:**
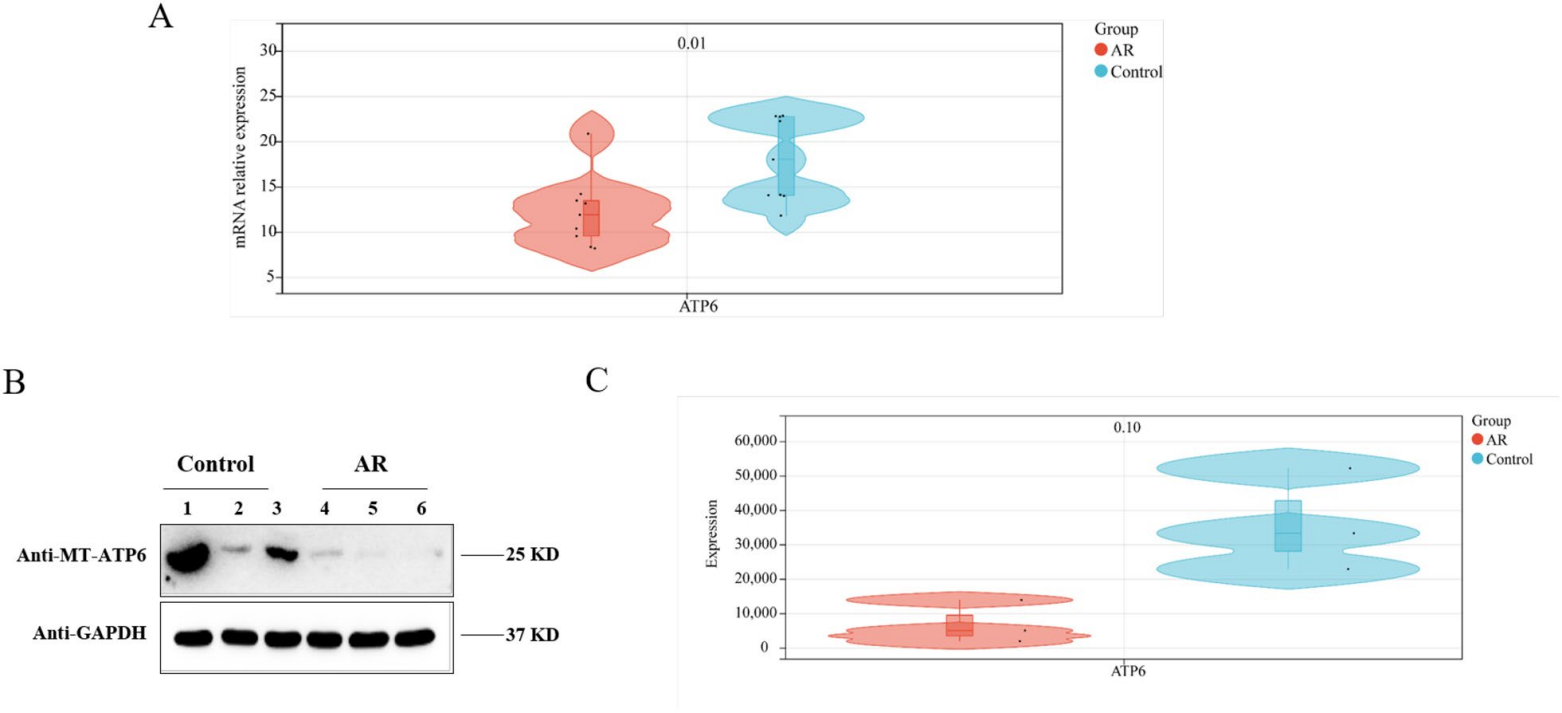
Comparison of the relative expression of mitochondrial genes between AR cases and control group. **A** Comparison of mitochondrial gene mRNA relative expression between AR and control groups (N = 9); **B**, **C** Comparison of gray scale diagram of mitochondrial gene protein relative expression between AR cases and control groups (N = 6)

A comparison of the gender and age distribution between the two groups suggests that the differences were not statistically significant (χ^2^ = 0, P = 1; t = 0.938, P = 0.362). Three patient pairs were matched for protein level evaluation. Figure 4A illustrates that the RNA levels of MT-ATP6 were significantly lower in AR individuals than in the controls (P < 0.05). Furthermore, a consistent trend was observed at the protein level, although the differences were not significant (Fig. 4B, C, P > 0.05).

## Discussion

New high-throughput genetic research techniques have revealed previously unknown mechanisms, such as mitochondrial genome variations, that may affect the likelihood of developing AR. This preliminary study explored the mitochondrial genome profile of AR in Chinese patients. Although the heteroplasmic level and SKAT analyses demonstrated no differences between groups, our two-phased approach identified a consistent association between AR and variant rs3135028 (MT8584.G/A) in MT-ATP6.

MT-ATP6 contributes to the rotational mechanism of ATP synthase as a component of the proton-transporting section of the enzyme [20]. Nonsynonymous variant rs3135028 (MT8584.G/A) in the exonic region of ATP6 resulted in an amino acid substitution (Ala20Thr). Although no pathogenic evidence of rs3135028 was suggested using SIFT (https://sift.bii.a-star.edu.sg) [21], MutationTaster2 (http://www.mutationtaster.org) [22], Polyphen-2 (http://genetics.bwh.harvard.edu/pph2/) [23], and PROVEAN (http://provean.jcvi.org) [24], this change from a non-polar, hydrophobic alanine to an uncharged, polar threonine may affect ATP6’s capacity to pump protons efficiently, reducing the amount of *ATP* energy units produced. In addition, we observed decreased levels of ATP6 mRNA and protein expression in nasal mucosal tissue from AR patients, suggesting that the variant may also affect the function of the enzyme through a dosage effect. Considering the small sample size of the expression assay, there may be false positives, and further validation with a larger size is required.

Also noteworthy were the gene-based suggestive associations found between two genes of interest (MT-RNR2 and MT-ND6) and AR. The MT*-*ND6 gene is an essential subunit of the mitochondrial membrane respiratory chain NADH dehydrogenase and a crucial component of the minimum assembly needed for catalysis. It links the flow of electrons to the pumping of protons and transferring electrons from NADH into the respiratory chain. Previously, rs28357671, located on the MT-ND6 gene, was associated with atopic dermatitis (AD) and asthma [16]. However, disease correlations at the genetic level have not been reported. In our study, seven nonsynonymous variants were identified in the discovery cohort. While three of the 68 AR cases carried variants, 10 out of 66 control individuals were identified as variants carriers. Analysis of the MT-RNR2 gene that encodes the 16S mitochondrial ribosomal RNA sequences revealed 36 variants, Six of which had a PhastCons100way vertebrate conservative prediction above 0.54 [25], suggesting possible deleterious effects of these variants on RNA function (Additional file 3: Table S3).

Due to the limited sample size in this study, genes associated with AR were likely overlooked because we lacked the statistical strength to identify weaker associations. It is also possible that some of the associations found in this analysis result from population structure artifacts. However, this is improbable given the demonstration that there is no apparent bias between the cases and controls.

## Conclusions

In summary, this study was the first to investigate mitochondrial gene profiles of Chinese patients with AR. Our results suggest that a common variant in ATP-6 and AR may be associated.

### Supplementary Information

**Additional file 1: Table S1.** PCR primers for SNaPshot analysis corresponding to mitochondrial genes.

**Additional file 2: Table S2.** PCR primers for qRT-PCR corresponding to mitochondrial genes.

**Additional file 3: Table S3.** Variants annotation for ND6 and RNR2.

**Additional file 4: Figure S1.** Association analysis of mtSNVs with AR.

## Data Availability

The datasets used and/or analysed during the current study are available from the corresponding author on reasonable request.

## Abbreviations

AR: Allergic rhinitis
MT: Mitochondrial
SKAT: Sequence kernel association tests
GWAS: Genome-wide association studies
SPT: Skin prick test
EDTA: Ethylenediaminetetraacetic acid
SNP: Single nucleotide polymorphisms
TPE: Total Protein Extraction
PVDF: Polyvinylidene difluoride
IQR: Interquartile range
