# Supplementary material for: Two-stage association study of mitochondrial DNA variants in allergic rhinitis
Source: Allergy Asthma Clin Immunol. 2024 Feb 23;20:16. doi: 10.1186/s13223-024-00881-z (PMC10893604; doi:10.1186/s13223-024-00881-z)
Supplement: Supplementary file 1 — Additional file 1: Table S1. PCR primers for SNaPshot analysis corresponding to mitochondrial genes. [file 13223_2024_881_MOESM1_ESM.docx]

**Table S1** PCR primers for SNaPshot analysis corresponding to mitochondrial genes

| **SNP NO.** | **F-seq（5’-3’）** | | **R-seq（5’-3’）** |
| --- | --- | --- | --- |
| rs1970771_rs28358875 | | CAATATGAAATGATCTGCTGCAGTG | CATCGGGGTAGTCYGAGTAACGTC |
| rs28357370 | | TTCCACCCWTACTACACAATCAAAGAC | GTATAGTAYGGATGCTACTTGTCCAATGAT |
| rs28357976_rs386419995_rs386419948 | | CAAGCAACYGCATCCATAATCCTTC | CTTGTTTCAGGTGCGAGATAGTAGTAGG |
| rs28358884_rs3135028 | | AGTGAAATGCCCCAACTAAATACTACC | GTTTTGAGGTTAGTTTGATTAGTCATTG |
| rs28380140_rs372078920 | | CCTCTCAGCCCTYCTAATGACCTC | TGGTGAGCTCAGGTGATTGATACT |
| rs2853517 | | AACAGTCACCCCYCAACTAACACATTA | CTGCATTGYTGCGTGCTTGA |
| rs28617389 | | CCCTTATTTACCRAGAAAGCTCAC | GTGGGGAAGAGRCTGATAATAAAGGT |
| rs28693675_rs373855397_rs148377232_rs34799580 | | TGGGTRCCACCCAAGTATTGACT | ATTGATTTCACGGAGGATGGTG |
| rs376513041 | | TAACCATCTTCTCCTTACACCTAGCAG | GGTGGGGATAGCGATGATTATG |
| rs376884056 | | CGCCTAATYTACTCCACCTCAATCAC | AGTRGYTGATTTGCGTTCAGTTGATG |
| rs386420001 | | CGTAGGAATTATATCCTTCTTGCTCATC | AGCGCCTAAGCATAGTGTTAGAGTTTG |
| rs386420019_rs28357678 | | CCATGCYTCAGGATACTCCTCAAT | TTCATCATGYGGAGATGTTGGATG |
| rs386829010 | | CCCYACCACACATTCGAAGAAC | TAGGGGAWGTAGCGTCTTGTAGA |
| rs386829183_rs386420024 | | ACTTCAACCTCCCTCACCATTG | GGGTAGAATCCGAGTATGTTGGAGAA |
| rs41467651_rs200487531 | | TAATCAACACCCTCCTAGCCTTACTAC | TTCGCAGGCGGCAAAGRCTA |

Abbreviations: SNP, single nucleotide polymorphism.
