# Supplementary material for: Two-stage association study of mitochondrial DNA variants in allergic rhinitis
Source: Allergy Asthma Clin Immunol. 2024 Feb 23;20:16. doi: 10.1186/s13223-024-00881-z (PMC10893604; doi:10.1186/s13223-024-00881-z)
Supplement: Supplementary file 2 — Additional file 2: Table S2. PCR primers for qRT-PCR corresponding to mitochondrial genes. [file 13223_2024_881_MOESM2_ESM.docx]

**Table S2** PCR primers for qRT-PCR corresponding to mitochondrial genes

| **Name** | **Sequence (5’->3’)** |
| --- | --- |
| MT-ND2-F | AGCATACTCCTCAATTACCCACA |
| MT-ND2-R | AGGTGCGAGATAGTAGTAGGGT |
| MT-ATP6-F | TAGCCCACTTCTTACCACAAGGCA |
| MT-ATP6-R | TGAGTAGGTGGCCTGCAGTAATGT |
| GAPDH-F | GTCTCCTCTGACTTCAACAGCG |
| GAPDH-R | ACCACCCTGTTGCTGTAGCCAA |
