# Supplementary material for: Two-stage association study of mitochondrial DNA variants in allergic rhinitis
Source: Allergy Asthma Clin Immunol. 2024 Feb 23;20:16. doi: 10.1186/s13223-024-00881-z (PMC10893604; doi:10.1186/s13223-024-00881-z)
Supplement: Supplementary file 3 — Additional file 3: Table S3. Variants annotation for ND6 and RNR2. [file 13223_2024_881_MOESM3_ESM.docx]

**Table S3** Variants annotation for ND6 and RNR2

| **Gene** | **Position** | **Ref Allele** | **Alt Allele** | **Gene Region** | **Function** | **PhastCons**  **100way** |
| --- | --- | --- | --- | --- | --- | --- |
| ND6 | 14162 | G | A | exonic | nonsynonymous SNV | 0 |
| ND6 | 14178 | T | C | exonic | nonsynonymous SNV | 0 |
| ND6 | 14180 | T | C | exonic | nonsynonymous SNV | 0.338583 |
| ND6 | 14318 | T | C | exonic | nonsynonymous SNV | 0 |
| ND6 | 14319 | T | C | exonic | nonsynonymous SNV | 0 |
| ND6 | 14418 | C | T | exonic | nonsynonymous SNV | 0 |
| ND6 | 14502 | T | C | exonic | nonsynonymous SNV | 0.00787402 |
| RNR2 | 1673 | T | C | ncRNA_exonic |  | 0 |
| RNR2 | 1694 | T | C | ncRNA_exonic |  | 0 |
| RNR2 | 1709 | G | A | ncRNA_exonic |  | 0 |
| RNR2 | 1709 | G | T | ncRNA_exonic |  | 0 |
| RNR2 | 1715 | C | T | ncRNA_exonic |  | 0 |
| RNR2 | 1721 | C | T | ncRNA_exonic |  | 0 |
| RNR2 | 1734 | C | T | ncRNA_exonic |  | 0 |
| RNR2 | 1736 | A | G | ncRNA_exonic |  | 0 |
| RNR2 | 1811 | A | G | ncRNA_exonic |  | 0 |
| RNR2 | 1824 | T | C | ncRNA_exonic |  | 0 |
| RNR2 | 1896 | T | C | ncRNA_exonic |  | 0 |
| RNR2 | 1978 | A | G | ncRNA_exonic |  | 0 |
| RNR2 | 2059 | C | T | ncRNA_exonic |  | 0 |
| RNR2 | 2218 | C | T | ncRNA_exonic |  | 0 |
| RNR2 | 2222 | T | C | ncRNA_exonic |  | 0 |
| RNR2 | 2226-2226 | - | A | ncRNA_exonic |  | 0 |
| RNR2 | 2281 | A | G | ncRNA_exonic |  | 0 |
| RNR2 | 2404 | T | C | ncRNA_exonic |  | 0 |
| RNR2 | 2706 | A | G | ncRNA_exonic |  | 0 |
| RNR2 | 2766 | C | T | ncRNA_exonic |  | 0 |
| RNR2 | 2835 | C | T | ncRNA_exonic |  | 0 |
| RNR2 | 2885 | T | C | ncRNA_exonic |  | 0 |
| RNR2 | 3083 | T | C | ncRNA_exonic |  | 0 |
| RNR2 | 3144 | A | G | ncRNA_exonic |  | 0 |
| RNR2 | 3167-3167 | - | C | ncRNA_exonic |  | 0 |
| RNR2 | 3197 | T | C | ncRNA_exonic |  | 0 |
| RNR2 | 3204 | C | T | ncRNA_exonic |  | 0 |
| RNR2 | 3206 | C | T | ncRNA_exonic |  | 0 |
| RNR2 | 3221 | A | G | ncRNA_exonic |  | 0 |
| RNR2 | 1719 | G | A | ncRNA_exonic |  | 0.015748 |
| RNR2 | 2356 | A | G | ncRNA_exonic |  | 0.543307 |
| RNR2 | 2833 | A | G | ncRNA_exonic |  | 0.755906 |
| RNR2 | 3010 | G | A | ncRNA_exonic |  | 0.992126 |
| RNR2 | 3097 | T | G | ncRNA_exonic |  | 1 |
| RNR2 | 3098 | T | G | ncRNA_exonic |  | 1 |
| RNR2 | 3100 | T | G | ncRNA_exonic |  | 1 |

Gene, genes with mutation sites. Position, location on the corresponding chromosome. Ref Allele, alleles with loci in the reference sequence. Gene Region, where the mutation is located. Function, gene function. PhastCons100way, conservative annotation of phastCons100way database.
