# Supplementary figures and images for: Two-stage association study of mitochondrial DNA variants in allergic rhinitis
Source: Allergy Asthma Clin Immunol. 2024 Feb 23;20:16. doi: 10.1186/s13223-024-00881-z (PMC10893604; doi:10.1186/s13223-024-00881-z)

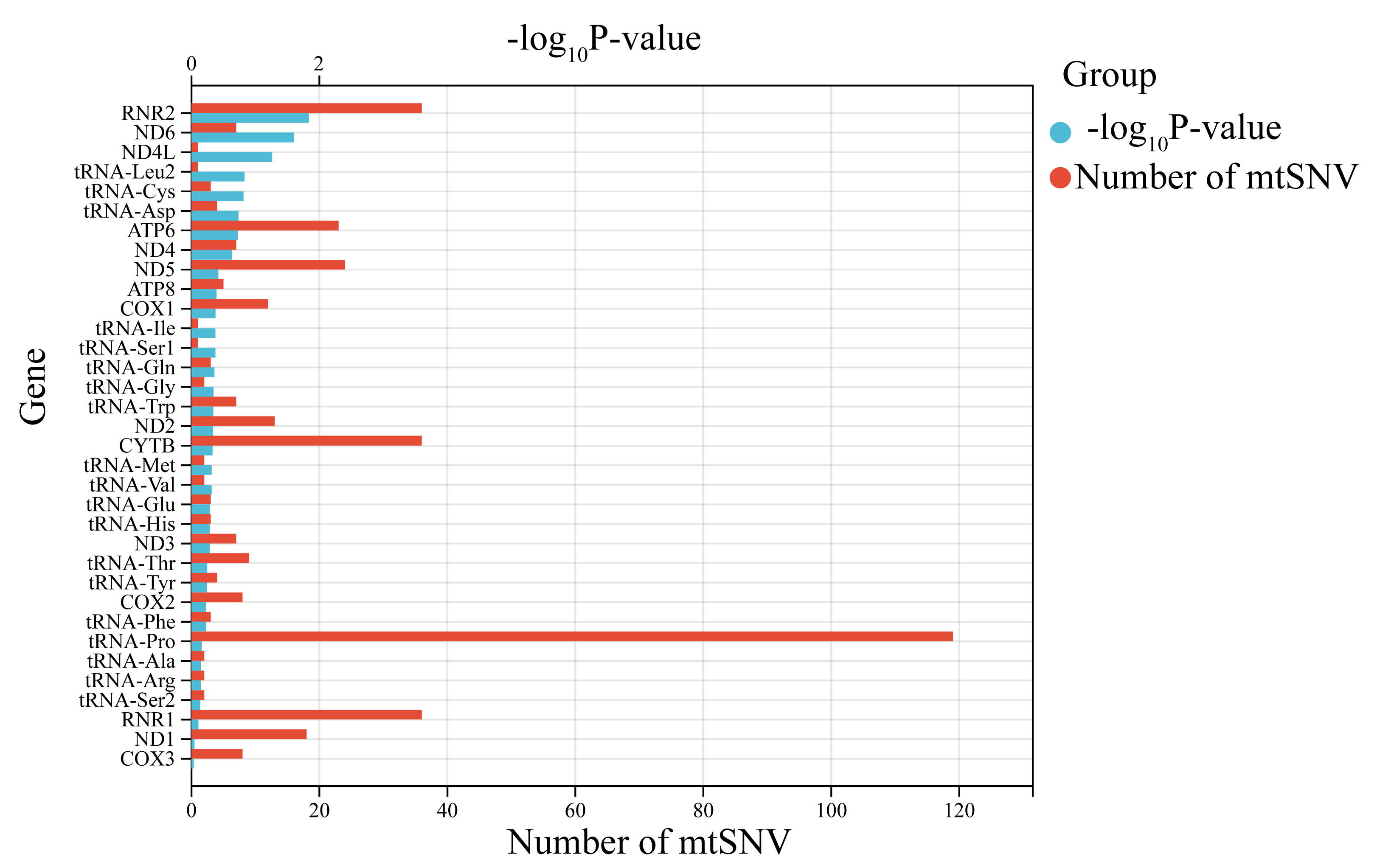

Supplement: Supplementary file 4 — Additional file 4: Figure S1. Association analysis of mtSNVs with AR. [file 13223_2024_881_MOESM4_ESM.docx]
